# Increased suicide attempt risk in people with epilepsy in the presence of concurrent psychogenic nonepileptic seizures

**DOI:** 10.1136/jnnp-2022-329093

**Published:** 2022-06-21

**Authors:** Irene Faiman, John Hodsoll, Allan H Young, Paul Shotbolt

**Affiliations:** 1 Department of Psychological Medicine, King’s College London Institute of Psychiatry Psychology and Neuroscience, London, UK; 2 Maudsley Hospital, South London and Maudsley NHS Foundation Trust, London, UK

**Keywords:** epilepsy, conversion disorder, paroxysmal disorder, psychiatry, neuropsychiatry

## Abstract

**Objectives:**

To test the hypothesis that people with concurrent diagnosis of epilepsy and psychogenic nonepileptic seizures (PNES) are at increased risk of attempting suicide as compared to people with epilepsy or PNES alone. To report on suicide rates.

**Methods:**

Retrospective cohort study from the UK largest tertiary mental health care provider, with linked nationwide admission and mortality data from the Hospital Episode Statistics and Office for National Statistics. Participants were 2460 people with a primary or secondary diagnosis of epilepsy, PNES or concurrent epilepsy and PNES attending between 1 January 2007 and 18 June 2021. The primary outcome was a first hospital admission for suicide attempt (International Classification of Diseases, version 10 X60–X84).

**Results:**

9% of participants had at least one suicide attempt-related hospital admission. For people with concurrent diagnosis of epilepsy and PNES, the odds for suicide attempt-related admissions were 2.52 times the odds of people with epilepsy alone (OR 0.40; 95% CI 0.21 to 0.79; p=0.01). Odds were comparable between people with concurrent diagnosis and people with PNES alone (OR 0.75; 95% CI 0.41 to 1.48; p=0.40). Post hoc analyses revealed that the odds of people with PNES alone were 1.93 times the odds of people with epilepsy alone (OR 0.52; 95% CI 0.38 to 0.70; p<0.001).

**Conclusions:**

People with concurrent diagnosis of epilepsy and PNES or PNES alone have significantly increased odds of hospitalisation due to suicide attempt as compared to people with epilepsy alone (152% and 93% increase, respectively). These findings have direct implications for the clinical management of suicide risk in people with epilepsy.

**WHAT IS ALREADY KNOWN ON THIS TOPIC**

- Suicide is three times more frequent in people with epilepsy and particularly in the presence of comorbid psychiatric disease.
- Approximately 12% of people with epilepsy have concurrent psychogenic nonepileptic seizures (PNES).
- To improve suicide prevention strategies, it is essential to quantify the risk imposed by concurrent PNES on suicide attempts in people with epilepsy.

**WHAT THIS STUDY ADDS**

- In the presence of concurrent PNES, the odds of people with epilepsy to be hospitalised for a first suicide attempt are 152% higher than when epilepsy occurs in isolation.

**HOW THIS STUDY MIGHT AFFECT RESEARCH, PRACTICE AND/OR POLICY**

- These findings have direct implications for clinical management of suicide risk for people with epilepsy.

## Introduction

Suicide results in over 800 000 deaths per year globally, and it represents the second most common cause of death in people between 15 and 29 years, and the fifth in people aged 30–49 years.^1^ The WHO calls for suicide prevention to become a global and national health priority, including implementation of strategies at national level as well as identification of high-risk groups for targeted psychosocial interventions.^1^

Epilepsy is one of the most common neurological disorders worldwide. It is estimated that in people with epilepsy, suicide is at least three times more frequent than in the general population.^2^ An accurate assessment of suicide risk in people with epilepsy is therefore a clinical and national health priority.

A recent meta-analysis reports that a substantial proportion (12%) of people with epilepsy have comorbid psychogenic nonepileptic seizures (PNES).^3^ These are episodes of abrupt paroxysmal change in behaviour or consciousness in the absence of the neuro-electrophysiological changes that accompany epileptic seizures.^4^ A diagnosis of a dissociative disorder such as PNES is associated with higher rates of suicide attempts.^5^ A previous suicide attempt has been identified as the single most important risk factor for suicide.^1^ Recognition and management of psychiatric disorders including PNES in people with epilepsy should therefore play a crucial role in suicide prevention.

Limited data and lack of community-level studies on people with concurrent diagnosis of epilepsy and PNES^6^ pose considerable challenges towards accurate clinical risk assessment.

To our knowledge, this is the first study quantifying the risk imposed by concurrent PNES on suicide attempts in people with epilepsy. We interrogated a large and extensive patient cohort (n=2460) to test the hypothesis that people with a concurrent diagnosis of epilepsy and PNES are at increased risk of attempting suicide as compared with people with epilepsy alone or PNES alone. Through this hypothesis testing study, we aimed to disentangle the individual effect of PNES and that of epileptic seizure disorder on the risk of attempting suicide. We provide evidence that can be directly and intuitively applied to risk assessment of patient groups in clinical practice.

## Methods

### Design and setting

This is a retrospective cohort study. Data were sourced from patients attending the South London and Maudsley Hospital (SLaM) between 1 January 2007 and 18 June 2021. SLaM is the largest tertiary mental healthcare provider in Europe, serving four south London boroughs with a total population of over 1.2 million people.^7^ It is the only NHS national specialist tertiary service in the UK offering treatment for people with severe dissociative disorders, including PNES. Data from the SLaM electronic clinical record system were extracted in deidentified format by a research nurse using the Clinical Record Interactive Search (CRIS).^7^

### Study population

We included people with a primary or secondary diagnosis of epilepsy (International Classification of Diseases, version 10 (ICD-10) code G40) and/or PNES (ICD-10 F44.5). In order to minimise the influence of comorbid structural brain disease, which is the comorbidity associated with highest risk of all-cause mortality in people with epilepsy,^8^ we excluded people with a primary or secondary diagnosis of cerebral malignancy (ICD-10 C71, C79.3, D43.0–D43.2, D49.6), traumatic brain injury (ICD-10 S01, S02, S06, S07, S09), dementia or progressive neurodegenerative disease (ICD-10 F00–F03). We excluded people with a primary or secondary diagnosis of psychotic disorder (ICD-10 F20–F29), as the robust association with suicide attempt rates in this population^9^ is likely to represent a phenomenologically and neurobiologically distinct phenomenon.^10 11^

### Suicide and suicide attempts

Data on admissions for suicide attempts were obtained from Hospital Episode Statistics (HES) admission data linked to our patient sample. HES includes routine hospital data collected at the national level, including information on all NHS admissions for suicide attempt registered throughout the UK (http://www.hscic.gov.uk/hes). We defined people with a registered admission under ICD-10 codes X60–X84 as cases of suicide attempt. Only information on the first presentation with suicide attempt was extracted. In line with CRIS data access permissions, HES data were extracted for all patients included in our cohort, except for those who at the time of the study were under 18, and for those that were only ever seen in SLaM while under the age of 18.

Mortality data (date and ICD-10 codes for cause of death) were obtained from the Office for National Statistics (ONS) data linked to our patient sample. We defined people with a registered ICD-10 primary code of X60–X84 at date of death as cases of confirmed suicide.

### Control variables

Control variables included age at first presentation with suicide attempt, ethnicity, gender, presence of other psychiatric diagnoses prior to suicide attempt, antidepressant prescription in the year prior to suicidal attempt, first instance of reported suicidal ideation prior to suicide attempt, and living alone prior to suicide attempt.

Age at first instance of suicide attempt was the age difference between date of suicide attempt and date of birth; in the absence of admission for suicide attempt, the age reported was the age at data extraction, that is, 18 June 2021, or age at death if deceased. Ethnicity groupings were based on the ONS 2011 Census ethnic group recommended categorisation,^12^ and further divided into White ethnicity, Other ethnicity (including Asian, Black, Mixed, Multiple and Other) and Unknown ethnicity. Information on whether the following disorders were recorded before the date of suicide attempt (or at any time if suicide attempt was not observed) was extracted: Bipolar Disorder (ICD-10 F31), Depressive disorders (ICD-10 F32–F33), Anxiety and stress-related disorders (including Obsessive–Compulsive Disorder and Post-Traumatic Stress Disorder; ICD-10 F40–F42, F43.1), Substance Misuse Disorders (ICD-10 F10–F19), Personality Disorder (ICD-10 F60), Developmental Disorders or mental retardation (ICD-10 F71–F73, F84).

Information on antidepressant drug prescription in the year prior to suicide attempt (or at any time if suicide attempt was not observed) was extracted using the CRIS embedded Medication App. Antidepressants were drugs listed under section 4.3 in the British National Formulary,^13^ with the addition of lithium. The Medication App retrieves a combination of structured information from medication and pharmacy prescription forms, and unstructured information from free-text notes and correspondence using Natural Language Processing (NLP) and has 85% precision and 75% recall for active prescription of antidepressants. First instances of suicidal ideation and a history of living alone prior to suicide attempt (or at any time if suicide attempt was not observed) were also extracted using CRIS embedded NLP apps. The NLP Lives Alone app has 77% precision and 83% recall for mentions of living alone. The NLP Suicidal Ideation app has 81% precision and 87% recall to instances of suicidal ideation. We further reviewed the performance of the NLP Suicidal Ideation app in our sample by inspecting all text snippets resulting in a positive instance of suicidal ideation (n=616) and calculated a 71% recall to instances of suicidal ideation occurring at any time before the snippet date. All false positives were subsequently corrected.

### Statistical analyses

**Figure 1:**
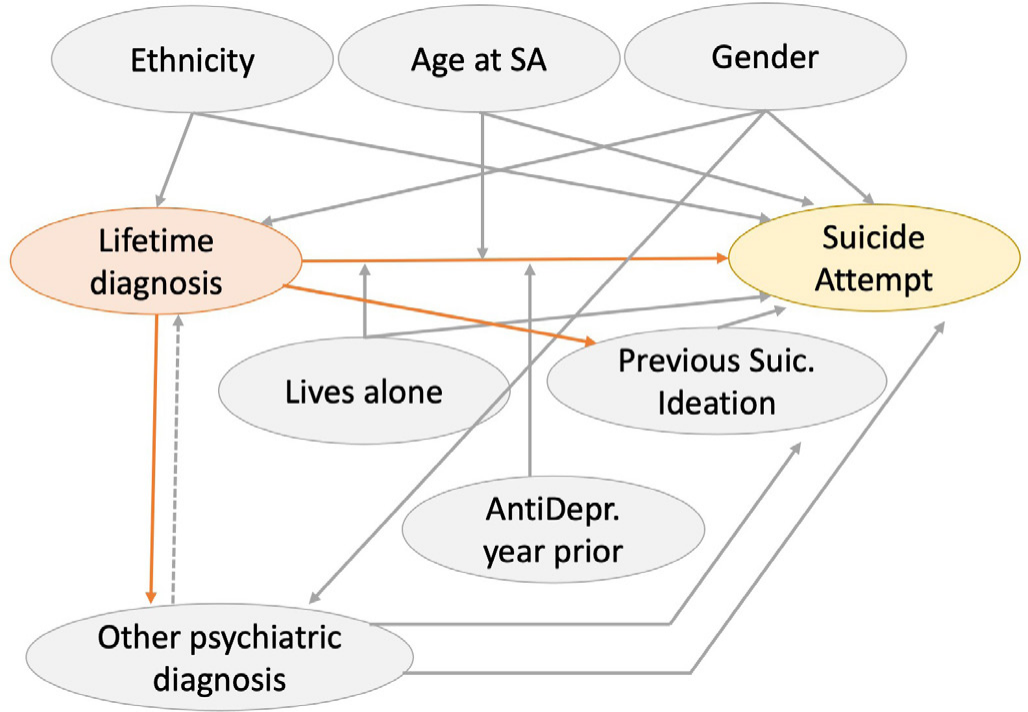
Causal diagram depicting variable relationships. An arrow’s direction represents the theoretical direction of causal effect. An arrow pointing to the middle body of another arrow indicates a theoretical moderation effect. The dashed arrow represents a potential confounding effect excluded by control analyses. Orange: independent variable. Yellow, dependent variable. Grey, control variables. AntiDepr., Antidepressant prescription; Suic., suicidal. SA, suicide attempt.

We tested the hypothesis that people with a concurrent diagnosis of epilepsy and PNES have higher odds of attempting suicide as compared with people with epilepsy alone or PNES alone. A causal diagram describing the relationship between study variables was built by a consultant neuropsychiatrist and a consultant psychiatrist (PS and AY) based on clinical and academic expertise (figure 1). In accordance with recommended strategies for accurate estimation of total effects,^14^ the causal diagram was used to guide variable entry in a Firth’s Bias-Reduced Logistic Regression model with penalised log-likelihood estimator.^15^ The Firth’s method is a penalised logistic regression approach used to obtain nearly unbiased maximum likelihood estimates in the presence of small case numbers or rare events leading to low cell count.^16 17^ The Firth’s method has been shown to produce reliable confidence intervals and log Odds Ratios (ORs) when the rarer outcome (here, suicide attempt) is supported by a small number of cases (n=12 in the concurrent diagnosis group).^17^ We tested whether an association exists between lifetime diagnosis and first instance of suicide attempt. The group with concurrent diagnosis of epilepsy and PNES was set as the reference category. Gender and ethnicity were included in the model as covariates.^14^ ORs and associated Confidence Intervals were derived to estimate the magnitude of the association. The change in odds between people with epilepsy alone and people with PNES alone was explored in post-hoc analyses. In our causal diagram, we propose that having other psychiatric diagnoses works as a mediator of the effect of lifetime diagnosis on suicide attempt-related admissions (figure 1). However, to exclude the possibility of a confounding effect of other psychiatric diagnoses on lifetime diagnosis (figure 1, dashed line), control analyses were conducted also adjusting for each comorbid psychiatric condition individually. We additionally performed unadjusted analyses exploring the association between lifetime diagnosis and first instance of suicide attempt in the absence of any model covariates. All people for whom data were collected were included in the analyses. Data were analysed using R statistical software^18^ V.4.0.0 (24 April 2020) and the ‘logistf’ package^19^ V.1.24 (10 September 2020). Deaths by suicide were calculated from ONS data at the whole sample level and individually per each diagnostic group as the number of confirmed deaths by suicide divided by the total number of deaths.

## Results

A total of 2460 people met the inclusion criteria and were included in the study.

### Deaths by suicide

Deaths by suicide accounted for 1.23% (2/163) of all deaths. Deaths by suicide were 0.85% (1/118) in the epilepsy group, and 2.63% (1/38) in the PNES group. No estimate could be provided for the group of people with concurrent diagnosis of epilepsy and PNES due to low numbers (0 deaths by suicide in 7 deaths).

### Suicide attempts

Overall, 9% (223/2460) of the individuals had at least one suicide attempt leading to hospital admission. Demographic and clinical characteristics of the included sample are reported in table 1.

**Table 1:** Demographic and clinical characteristics of 2460 people seen at SLaM between 1 January 2007 and 18 June 2021

	All	No suicide attempt	Suicide attempt
Total	2460 (100)	2237 (90.9)	223 (9.1)
Lifetime diagnosis			
Epilepsy	1343 (54.6)	1261 (93.9)	82 (6.1)
PNES	1040 (42.3)	911 (87.6)	129 (12.4)
Concurrent diagnosis	77 (3.1)	65 (84.4)	12 (15.6)
Gender			
Male	1027 (41.8)	956 (93.1)	71 (6.9)
Female	1433 (58.2)	1281 (89.4)	152 (10.6)
Ethnicity			
White	1578 (64.1)	1404 (89.0)	174 (11.0)
Other	520 (21.2)	496 (95.4)	24 (4.6)
Unknown	362 (14.7)	337 (93.1)	25 (6.9)
Age at suicide attempt (if no SA, age at data extraction or death)			
Age, mean (SD)	38.8 (17.5)	39.6 (17.7)	30.6 (12.9)
Other psychiatric diagnoses prior to SA (if no SA, at any time)			
Depression disorders / Bipolar Disorder			
No	2104 (85.5)	1916 (91.1)	188 (8.9)
Yes	356 (14.5)	321 (90.2)	35 (9.8)
Anxiety and stress-related disorders			
No	2162 (87.9)	1950 (90.2)	212 (9.8)
Yes	298 (12.1)	287 (96.3)	11 (3.7)
Substance Misuse Disorders			
No	2356 (95.8)	2140 (90.8)	216 (9.2)
Yes	104 (4.2)	97 (93.3)	7 (6.7)
Personality Disorder			
No	1990 (80.9)	1775 (89.2)	215 (10.8)
Yes	470 (19.1)	462 (98.3)	8 (1.7)
Developmental Disorder / Intellectual Disability			
No	2396 (97.4)	2178 (90.9)	218 (9.1)
Yes	64 (2.6)	59 (92.2)	5 (7.8)
Previous suicidal ideation (if no SA, at any time)			
No	2091 (85.0)	1888 (90.3)	203 (9.7)
Yes	369 (15.0)	349 (94.6)	20 (5.4)
Lives alone prior to SA (if no SA, at any time)			
No	2190 (89.0)	1976 (90.2)	214 (9.8)
Yes	270 (11.0)	261 (96.7)	9 (3.3)
Antidepressant prescription in the year prior to SA (if no SA, at any time)			
No	1126 (45.8)	930 (82.6)	196 (17.4)
Yes	1334 (54.2)	1307 (98.0)	27 (2.0)

Reported are number of cases (percentages) unless otherwise specified.

PNES, psychogenic nonepileptic seizures; SLaM, South London and Maudsley Hospital.

**Figure 2:**
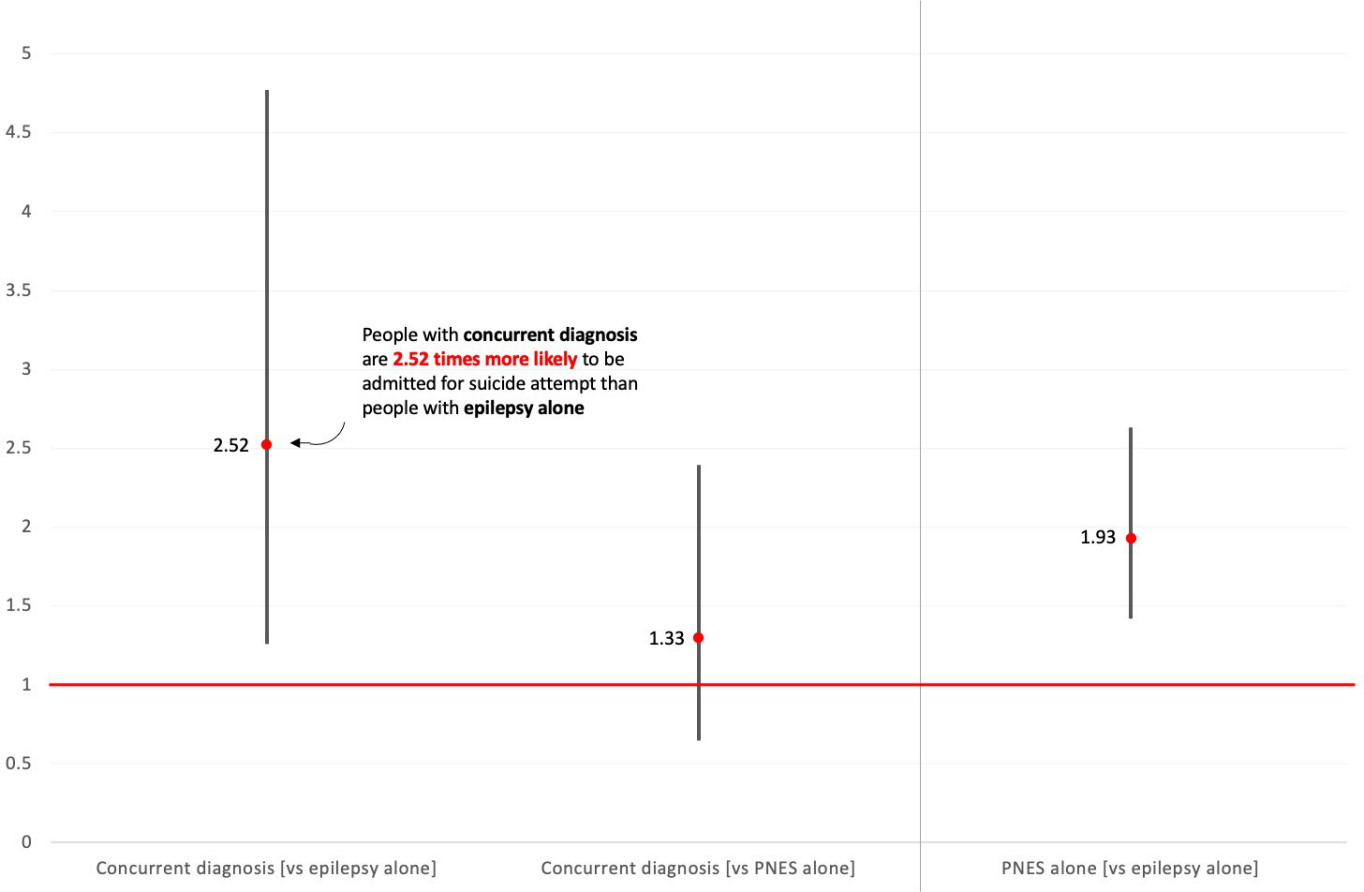
Summary of results. Expressed as 1/OR for visualisation and interpretation purposes, with 95% CIs. PNES, psychogenic nonepileptic seizures.

The Firth’s Bias-Reduced logistic regression analysis revealed that there is a significant change in odds of being admitted for suicide attempt between people with epilepsy alone and people with concurrent diagnosis (adjusted OR 0.40; 95% CI 0.21 to 0.79; table 2). The odds for people with PNES alone and people with concurrent diagnosis are comparable (adjusted OR 0.75; 95% CI 0.41 to 1.48; table 2). In other words, the odds of people with concurrent diagnosis to have a first suicide attempt-related admission are 1/0.40=2.52 times the odds of people with epilepsy alone (and this change in odds is significant), and 1/0.75=1.33 times the odds of people with PNES alone (and this change in odds is not significant; figure 2).

**Table 2:** Results from Firth’s Bias-Reduced logistic regression analysis on admission for suicide attempt

	B (SE)	OR	95% CI for OR (lower)	95% CI for OR (upper)	P value
**Against concurrent diagnosis of epilepsy and PNES**					
Unadjusted*					
Epilepsy	−1.07 (0.33)	0.34	0.18	0.68	<0.01
PNES	−0.29 (0.32)	0.74	0.41	1.46	0.37
Adjusted†					
Epilepsy	−0.94 (0.33)	0.40	0.21	0.79	0.01
PNES	−0.27 (0.32)	0.75	0.41	1.48	0.40
Post hoc analysis: against PNES					
Unadjusted*					
Epilepsy	−0.77 (0.15)	0.46	0.34	0.61	<0.001
Adjusted†					
Epilepsy	−0.66 (0.15)	0.52	0.38	0.70	<0.001

Presented as beta values (B) and their Standard Errors (SE), Odds Ratios (OR) with 95% Confidence Intervals (CI), and significance value (p value).

*Unadjusted model includes lifetime diagnosis as the only model predictor, without any additional covariates.

†Adjusted for gender and ethnicity. The ORs are the total effect of lifetime diagnosis through all causal pathways including age, previous suicidal ideation, other psychiatric diagnoses, lives alone and antidepressants prescription in the year before admission for suicide attempt.

PNES, psychogenic nonepileptic seizures.

Results of the post hoc analysis revealed that there is a significant change in odds of being admitted for suicide attempt between people with epilepsy alone and people with PNES alone (adjusted OR 0.52; 95% CI 0.38 to 0.70; table 2). In other words, the odds of people with PNES alone to have a first admission for suicide attempt are 1/0.52=1.93 times the odds of people with epilepsy alone (figure 2).

These OR estimates represent the total effect of lifetime diagnosis on suicide attempt-related admissions, that is, the net effect of all associations of lifetime diagnosis through all causal pathways to suicide attempt, at any category of gender and ethnicity (figure 1).^14^ Results from the unadjusted analyses revealed similar findings (table 2). Control analyses excluded the possibility that any of the psychiatric comorbidities under study were determinant drivers of the observed associations (table 3).

**Table 3:** Results from the control analyses on admission for suicide attempt, adjusting for gender, ethnicity and each comorbid psychiatric condition

	B (SE)	OR	95% CI for OR (lower)	95% CI for OR (upper)	P value
Against concurrent diagnosis of epilepsy and PNES*. Controlling for:					
Depression disorders / Bipolar Disorder					
Epilepsy	−0.94 (0.33)	0.39	0.21	0.78	<0.01
PNES	−0.28 (0.32)	0.75	0.41	1.48	0.39
Anxiety and stress-related disorders					
Epilepsy	−0.90 (0.33)	0.41	0.22	0.82	0.01
PNES	−0.27 (0.32)	0.76	0.42	1.51	0.42
Substance Misuse Disorders					
Epilepsy	−0.94 (0.33)	0.39	0.21	0.78	<0.01
PNES	−0.28 (0.32)	0.75	0.41	1.49	0.40
Personality Disorder					
Epilepsy	−0.94 (0.33)	0.39	0.21	0.78	<0.01
PNES	−0.27 (0.32)	0.76	0.41	1.49	0.41
Developmental Disorder / Intellectual Disability					
Epilepsy	−0.70 (0.33)	0.49	0.26	0.99	0.04
PNES	−0.33 (0.32)	0.71	0.39	1.41	0.32
Post hoc analysis: against PNES*. Controlling for:					
Depression disorders / Bipolar Disorder					
Epilepsy	−0.66 (0.15)	0.52	0.38	0.70	<0.001
Anxiety and stress-related disorders					
Epilepsy	−0.63 (0.15)	0.53	0.39	0.72	<0.001
Substance Misuse Disorders					
Epilepsy	−0.66 (0.15)	0.52	0.38	0.70	<0.001
Personality Disorder					
Epilepsy	−0.66 (0.15)	0.51	0.38	0.69	<0.001
Developmental Disorder / Intellectual Disability					
Epilepsy	−0.37 (0.16)	0.69	0.50	0.94	0.01

Presented are beta values (B) from the Firth’s Bias-reduced logistic regressions and their Standard Error (SE), Odds Ratios (OR) with 95% Confidence Intervals (CI), and significance value (p value).

*All control analyses are also adjusted for gender and ethnicity. The ORs are the total effect of lifetime diagnosis through all causal pathways including age, previous suicidal ideation, lives alone and antidepressants prescription in the year before admission for suicide attempt.

PNES, psychogenic nonepileptic seizures.

Two percent of people on antidepressant prescription had a registered suicide attempt compared with 17.4% of those without a prescription (table 1). It should be noted that due to the time-locked nature of this NLP variable, for people with a suicide attempt the absence of a prescription either indicates that antidepressants were not prescribed, or that a medication review was not performed in our centre in the year preceding the attempt.

## Discussion

To the best of our knowledge, this is the first study quantifying the risk of attempting suicide associated to a lifetime diagnosis of concurrent epilepsy and PNES, as compared with epilepsy alone and PNES alone. We show that in the presence of concurrent PNES, the odds of people with epilepsy to be hospitalised for a first suicide attempt are 152% higher than when epilepsy occurs in isolation. For people with PNES alone the odds of attempting suicide are 93% higher than the odds of people with epilepsy alone, while people with PNES alone and people with a concurrent diagnosis are at comparable risk. These associations are observed also after controlling for the influence of comorbid psychiatric diagnoses. The estimates represent a measure of total effect,^14^ which is a clinically intuitive measure of the overall risk associated to the presence of a diagnosis and its accompanying clinical profile, for any gender and ethnicity.

These findings have relevant and direct implication for risk assessment and management in clinical practice. When assessing and managing suicide risk in people with epilepsy, clinicians should be aware of the additional risk imposed by the concurrent presence of PNES. Given the substantial proportion (12%) of PNES in people with epilepsy,^3^ we identify this as a high-risk group that should be a target for enhanced risk monitoring and preventive psychosocial interventions.

Our study highlights the need to identify and implement effective risk prevention methods; strategies such as improving recognition and management of PNES are crucial.^20^ Confirming the diagnosis of PNES is a long process and a clinical challenge, and misdiagnosis of PNES as epilepsy is common.^21^ It is therefore essential to investigate cases where there is a suspicion for coexisting seizure types^6^ and to acknowledge diagnostic uncertainty^22^; failing to do so could lead to underestimation of risk in a population that is already at elevated risk of suicide,^2^ and for whom prevention of premature mortality is a top priority.^23^

Our study design allowed us to disentangle the individual effect of PNES and that of epileptic seizure disorder on the risk of attempting suicide in people with concurrent diagnosis. While this group was at significantly increased risk when compared with people with epilepsy alone, the risk was similar to that of people with PNES alone. This pattern suggests that for people with a concurrent diagnosis, the added risk of attempting suicide is mainly due to the presence of PNES and related clinical profile. We hypothesise that an ability to dissociate might enable engagement with suicidal behaviour, or alternatively that a suicide attempt might be a severe reaction to the burden of living with dissociative symptoms. Concurrent diagnosis should therefore not to be regarded simply as a coexistence of different seizure types; but rather that it is associated with a different risk profile, approximating to that of people with PNES alone.

No previous study has examined whether in the presence of concurrent PNES, people with epilepsy are at increased suicide attempt risk. Previous evidence, however, highlights that in epilepsy, the highest suicide risk occurs in the presence of comorbid psychiatric disease.^24 25^ Our study corroborates these findings and for the first time estimates the magnitude of risk specific to the concurrent presence of PNES. Given the scarcity of studies examining people with concurrent diagnosis,^3 6^ our findings represent a substantial advancement towards a better understanding of the psychopathology and risk profile associated to this condition.

Our observation that people with PNES alone and people with concurrent diagnosis are at comparable suicide attempt risk align with those of a small-sampled study reporting no difference in the number of suicide attempts between these two populations.^26^ Our results reinforce these findings with evidence from a large and extensive cohort. Additionally, by including a comparison with a large sample of people with epilepsy alone, we estimate the contribution of different seizure types on suicide attempt risk.

A recent meta-analysis estimated that suicide attempts in epilepsy have a pooled prevalence of 7.4%,^27^ in line with observed rates (6.1%) in our sample. This is even higher in PNES (19%),^28^ in line with our observations (12.4%).

The risk of premature mortality is elevated in both PNES and epilepsy, with suicide being one of the leading causes.^8 29^ In our sample with epilepsy alone, deaths by suicide accounted for 0.85% of observed deaths. This rate falls in the confidence interval reported by a recent meta-analysis, which estimated a pooled suicide rate of 0.5% in epilepsy.^27^ In our sample with PNES alone, deaths by suicide accounted for 2.63% of observed deaths, consistent with other reports of elevated suicide mortality rates (0.80%–7.3%) in PNES.^29 30^ Deaths by suicide could not be estimated for the group of people with concurrent diagnosis due to the low number of deceased people in our sample. However, based on the pattern emerging from our findings, we would expect deaths by suicide in this group to approximate to the numbers observed in people with PNES alone.

This study has several strengths. We studied a large cohort (n=2460) who attended the UK largest tertiary mental health institution over the last 15 years. The sizeable sample allowed us to focus on a relatively rare event (suicide attempt) in a clinical population (people with confirmed concurrent diagnosis) which is generally hard to identify and study due to small numbers.

We used linked national admission (HES) and mortality (ONS) data. HES and ONS linkage ensured that suicide attempt-related hospitalisations and deaths by suicide for our sample could be obtained independently from hospital location at time of admission, as these data are collected at the national level.

However, by only examining HES admission data, we did not consider cases that were not admitted to a hospital bed following a suicide attempt. It is estimated that approximately 50% of all cases presenting to hospital following self-harm do not get admitted,^31^ and a large proportion of cases do not attend hospital following a suicide attempt.^32^ Additionally, HES data quality might be poor for some sites.^31^ Therefore, the frequencies of suicide attempt reported in this study are likely to be underestimates, and the risk estimates reported only relate to suicide attempts requiring inpatient care and treatment.

It is accepted that there is potential for a degree of bias in the diagnosis of the conditions investigated. Information on whether the diagnoses of epilepsy and PNES were reached following gold-standard video-electroencephalogram confirmation^33^ or clinically only was not available due to the study’s retrospective nature. The diagnosis of epilepsy by non-specialists has an estimated risk of misdiagnosis between 19% and 26%; the misdiagnosis risk is lower (between 4.6% and 5.6%) following specialist review.^21 34^ Our data were sampled from a centre offering specialist psychiatric and neuropsychiatric review, so we estimate the misdiagnosis rate to be in the lowest range. It also hosts the only UK national specialist tertiary service offering diagnosis and treatment for severe dissociative disorders including PNES, so we expect misdiagnosis rates for PNES to be minimal.

As our sample came from a tertiary mental health hospital, our cohorts are likely to be more complex than the general population with epilepsy, PNES or concurrent diagnosis. Future studies sampling patients not receiving psychiatric management, as well as cross-national studies will further improve risk estimates accuracy.

Our estimates of the association between lifetime diagnosis and suicide attempt are derived from a priori theoretical assumptions depicted in our causal diagram (figure 1). This was built to represent the most plausible description of the variables’ interaction. However, we do not exclude the possibility that results might differ under different assumptions. As this was a hypothesis testing study, we were bound to create a causal diagram in accordance with recommended strategies for accurate estimation of total effects.^14^ This was done to the best of our knowledge and based on the clinical and academic expertise of two senior authors. Additionally, by running a series of control analyses, we excluded the possibility that the observed associations were driven by the presence of psychiatric comorbidities.

By examining Odds Ratios, we report on associations observed at the group level. This does not necessarily imply that prediction is possible for personalised risk assessment at the level of single patients.^35^ Our discussion of risk should be seen in this context and refers to the need to pay particular attention to the management of specific patient groups.

## Conclusion

This study shows that people with concurrent diagnosis of epilepsy and PNES and people with PNES alone are at significantly increased risk of being admitted to hospital due to a suicide attempt as compared with patients with epilepsy alone (152% and 93% increase, respectively). These findings have direct implications for clinical management of suicide risk for people with epilepsy.

It is essential to investigate cases where there is a suspicion for coexisting seizure types, as a concurrent diagnosis of epilepsy and PNES is associated with a different risk profile and a significantly increased risk of hospitalisation due to suicide attempt. People with concurrent diagnosis of epilepsy and PNES are a high-risk category that should be target of enhanced risk monitoring and preventive psychosocial interventions.

10.1136/jnnp-2022-329093.supp1 Supplementary data

## Data Availability

Data may be obtained from a third party and are not publicly available. Access to Clinical Record Interactive Search (CRIS) data used for this study is regulated by the CRIS Oversight Committee and the ‘Oxfordshire C’ Research Ethics approval for secondary analysis of CRIS data (18/SC/0372). Data used for this research cannot be shared without prior approval from the CRIS Oversight Committee. Those interested should contact Robert Stewart (robert.stewart@kcl.ac.uk), CRIS academic lead.
